# Does Incident Solar Ultraviolet Radiation Lower Blood Pressure?

**DOI:** 10.1161/JAHA.119.013837

**Published:** 2020-02-28

**Authors:** Richard B. Weller, Yuedong Wang, Jingyi He, Franklin W. Maddux, Len Usvyat, Hanjie Zhang, Martin Feelisch, Peter Kotanko

**Affiliations:** ^1^ Center for Inflammation Research University of Edinburgh United Kingdom; ^2^ Department of Statistics & Applied Probability University of California ‐ Santa Barbara Santa Barbara CA; ^3^ Integrated Care Analytics Fresenius Medical Care North America Waltham MA; ^4^ Renal Research Institute New York NY; ^5^ Clinical & Experimental Sciences Faculty of Medicine, and Institute for Life Sciences Southampton General Hospital University of Southampton United Kingdom

**Keywords:** cardiovascular disease, cardiovascular health, hypertension, nitric oxide, renal disease, seasonal variation, Epidemiology, Race and Ethnicity, Primary Prevention

## Abstract

**Background:**

Hypertension remains a leading global cause for premature death and disease. Most treatment guidelines emphasize the importance of risk factors, but not all are known, modifiable, or easily avoided. Population blood pressure correlates with latitude and is lower in summer than winter. Seasonal variations in sunlight exposure account for these differences, with temperature believed to be the main contributor. Recent research indicates that UV light enhances nitric oxide availability by mobilizing storage forms in the skin, suggesting incident solar UV radiation may lower blood pressure. We tested this hypothesis by exploring the association between environmental UV exposure and systolic blood pressure (SBP) in a large cohort of chronic hemodialysis patients in whom SBP is determined regularly.

**Methods and Results:**

We studied 342 457 patients (36% black, 64% white) at 2178 US dialysis centers over 3 years. Incident UV radiation and temperature data for each clinic location were retrieved from the National Oceanic and Atmospheric Administration database. Linear mixed effects models with adjustment for ambient temperature, sex/age, body mass index, serum Na^+^/K^+^ and other covariates were fitted to each location and combined estimates of associations calculated using the DerSimonian and Laird procedure. Pre‐dialysis SBP varied by season and was ≈4 mm Hg higher in black patients. Temperature, UVA and UVB were all linearly and inversely associated with SBP. This relationship remained statistically significant after correcting for temperature.

**Conclusions:**

In hemodialysis patients, in addition to environmental temperature, incident solar UV radiation is associated with lower SBP. This raises the possibility that insufficient sunlight is a new risk factor for hypertension, perhaps even in the general population.

**Clinical Perspective**

**What Is New?**

- In a large North American cohort of chronic hemodialysis patients, incident solar ultraviolet radiation was found to be associated with lower systolic blood pressure, even after adjustment for environmental temperature, showing that human exposure to ultraviolet light contributes to blood pressure regulation and accounts for seasonal and geographical variation.

**What Are the Clinical Implications?**

- While this is an observational study, our findings are consistent with data showing reduced blood pressure and lower rates of cardiovascular disease in populations with higher sun exposure, and suggest that Vitamin D independent benefits of sunlight should be considered when assessing risk benefit ratios.
- Sunlight exposure appears to lower blood pressure; insufficient exposure to natural ultraviolet radiation and/or active avoidance of sunlight may be new risk factors for hypertension.

Hypertension is a leading global cause for premature disease and death, affecting >1 billion people worldwide. Its pathophysiology involves complex interactions of multiple genetic, behavioral/lifestyle‐related and environmental risk factors and social stressors with the endocrine, metabolic, autonomic nervous and cardiovascular systems. Without overt symptoms, hypertension can remain unrecognized and escape detection^1^ for considerable time during which the raised blood pressure (BP) inflicts end‐organ damage. Multiple pharmacological and interventional treatment options exist but not all are well tolerated, in particular by older patients. There is convincing evidence from a large body of epidemiological data and clinical trials that BP lowering is associated with reduced cardiovascular risk and mortality, especially from myocardial infarction and stroke.^2^, ^3^

The 2017 ACC/AHA Guidelines^4^ for the Prevention, Detection, Evaluation and Management of Hypertension redefined what constitutes a “normal BP” to <120 mm Hg systolic and <80 mm Hg diastolic, irrespective of age, sensibly focusing on systolic blood pressure (SBP) in older patients.^5^ While these aggressive diagnostic thresholds and treatment targets have not been adopted by the 2018 ESC/ESH Guidelines for the Management of Arterial Hypertension^6^ their impact is nevertheless likely to extend well beyond the practice of medicine in the United States and evoked lively discussions.^7^, ^8^, ^9^, ^10^, ^11^, ^12^, ^13^, ^14^ In agreement with the “call to action” by the 2016 Lancet Commission on Hypertension,^2^ most guidelines emphasize non‐pharmacological intervention options before initiation of/in combination with antihypertensive drug treatment. Risk factors such as poor diet, salt/alcohol intake, smoking, obesity, and physical inactivity are the focus of current interventions. However, long‐term compliance is a concern, not all factors (eg, aging, (epi)genetics) are modifiable, and environmental risks such as transportation noise and air pollution^15^ are not easily avoided in urban areas. Comparatively little effort has been spent on identifying beneficial environmental factors, but geographical and seasonal variations in BP may provide important clues.

Seasonal BP variation was first reported in ischemic heart disease patients in the early 1960s.^16^ BP and cardiovascular mortality in temperate countries of the Northern hemisphere show a marked seasonal trend, being higher in winter than summer.^17^ Both daylight length and ambient temperature correlate inversely with BP.^18^ Population BP also correlates inversely with latitude.^19^ Chronic hemodialysis patients are no exception to these associations^20^ but have a markedly increased cardiovascular mortality risk due to the extra burden of fluid overload, chronic inflammation and oxidative stress.^21^, ^22^ Thus adequate BP control is essential to improve their outcomes.^20^ Identifying an easily modifiable environmental modulator of BP would suggest lifestyle interventions that could improve cardiovascular health in this vulnerable patient cohort.

While epidemiological data suggest a role for sunlight in lowering BP, its mechanism of action is uncertain. The warmth of sunshine may contribute to but cannot account entirely for seasonal BP variation when measured in a temperature‐controlled doctor's office or at home. In 2010, we proposed that solar ultraviolet (UV) radiation may contribute to cardiovascular health by releasing nitric oxide (NO) from storage forms in the skin^23^ and later demonstrated that short UV exposures acutely lower BP^24^ in healthy human volunteers. The relatively modest number of individuals investigated in earlier epidemiological studies prevented UV radiation from being identified as a potential BP‐modulating variable, and limited information is available as to whether effects differ between different ethnicities. We therefore sought to test the hypothesis that incident solar radiation modulates BP in a very large and diverse cohort of chronic hemodialysis patients. We chose the hemodialysis setting because in these patients BP is routinely and frequently measured in a procedurally standardized fashion in vast numbers, across multiple environmentally different locations within a single healthcare set‐up. We are unaware of the availability of other such temporally and geographically extensive blood pressure data sets. Although our findings are most directly applicable to patients on hemodialysis, we believe that they raise important questions for environmental management of population BP more generally.

## Methods

### Participants and Blood Pressure Data

This observational study included 342 457 patients undergoing chronic hemodialysis in 2178 US Fresenius Medical Care facilities (see Figure S1 for location of dialysis centers and distribution of patient numbers) between January 2011 and December 2013. It was reviewed by the Western Institutional Review Board's Affairs Department and as it was deemed to meet the conditions for exemption under 45 CFR 46.101 (b)(4) the requirement for consent was waived. Because of the sensitive nature of the data collected for this study, requests to access the data set from qualified researchers trained in human subject confidentiality protocols may be sent to the Renal Research Institute at 315 East 62nd Street, 4th Floor | New York, NY 10065. Patients visited dialysis facilities on average 3 times per week and had their BP measured before each treatment by a standard protocol while sitting, using an automated device with an appropriate‐size pressure cuff around the upper arm positioned at heart level. On average at least 10 to 15 minutes lapsed between arrival at the facility and BP measurements. We used monthly averages of pre‐dialysis systolic blood pressures (SBP) as the response variable. These monthly averages were based on an average of 11.3 measurements. We analyzed systolic rather than diastolic BP as in dialysis patients the former is associated with patient outcomes.^25^ Demographic variables such as race, sex, age, catheter use, and monthly averages of body mass index, interdialytic weight gain, albumin, erythropoietin use, hemoglobin, serum sodium and potassium were used as covariates. A diagnosis of hypertension in the patient record was also used as a co‐variate as this corresponds to anti‐hypertensive medication use.

### Collection of UV and Temperature Data

Our goal was to investigate associations between SBP and UV, with adjustment for clinical covariates and ambient temperature. Since it was not feasible to determine personal exposures to UV radiation and temperature, we approximated these exposures using environmental data retrieved from public databases at matched locations. The 2178 facilities were divided into 1530 geographical locations according to their zip codes. For each location, we first computed hourly spectral irradiances (W/m^2^) at each wavelength from 280 to 400 nm using the tropospheric UV and visible radiation model from the US National Center for Atmospheric Research (NCAR) (http://cprm.acom.ucar.edu/Models/TUV/Interactive_TUV/), corresponding to >25 million hourly records for UVA and UVB for the entire observation period. We then computed hourly UVA and UVB as the summations of spectral irradiances over wavelength ranges 321 to 400 and 280 to 320 nm, respectively. Lastly, we computed summations of hourly UVA and UVB over each day to approximate the total exposure, and averages of daily UVA and UVB to calculate monthly averages. See Figures S2 and S3 for seasonal averages of outdoor exposure to UVA/UVB radiation by location.

Daily average temperatures (°C) for all locations were derived from the US National Oceanic and Atmospheric Administration web site (http://www.ncdc.noaa.gov/cdo‐web/search). See Figure S4 for average outdoor temperatures by location. For locations lacking temperature stations with matching latitude/longitude we approximated temperatures from those within the shortest great circle distance using spherical law of cosines.

### Statistical Analyses

Our aim was to investigate the relationship between pre‐dialysis SBP and UVA/UVB exposure. Since we had repeated measurements of SBP from each patient, associations may vary among different locations, and the data set was too large to fit a single spatial‐temporal model, we adopted a 2‐stage procedure. (1) We first fitted a linear mixed‐effects model for repeated measurements from all patients at each location with an unstructured covariance matrix for random intercept and slope. This model takes into account between‐patient variability and the within‐patient correlation of SBP measurements across time points. This approach produced estimates of population intercept and slope at each location. (2) We then computed a combined estimate of association based on estimated population slopes from all locations using the DerSimonian and Laird procedure for random effects meta‐analysis,^26^ including CIs and *P* values. To assess the assumption of the second stage analysis, we fitted thin‐plate splines to explore spatial patterns. We considered 3 analyses for associations between SBP and UVA/UVB: Model 1: no adjustment for covariates; Model 2: adjustments for sex, age, hypertension, use of central venous catheter as vascular access, body mass index, interdialytic weight gain, albumin, erythropoietin dose, hemoglobin, serum sodium, and potassium, and a linear trend over calendar time; Model 3: adjustment for ambient temperature in addition to all Model 2 covariates. As expected, incident UV radiation and environmental temperature were closely associated. To detect potential problems with collinearity, we computed the variance inflation factor of temperature when UVA or UVB was in the model. The variance inflation factors equaled 2.82 for the combination of temperature and UVA and 2.85 for temperature and UVB; since both were smaller than the cutoff value of 5, we deemed that collinearity was not a problem. Separate analyses were performed for black and white patients. We used the complete case analysis since we have a large data set, from which there was only a small percentage of missing values (Table S1). Other than age, most missing values were caused by missing dialysis treatments. This was typically because of patient hospitalization. We adopted the partly conditional approach where the analysis is conditional on being alive.^27^ To address concerns about correlation among estimated slopes we fitted a thin‐plate spline model to slope estimates to investigate spatial pattern. Based on these fits we concluded that correlation of the slope estimates across sites was negligible relative to the amount of noise in them. As a sensitivity analysis, we divided all patients into 4 groups according to longitude and 4 groups according to latitude. Estimates based on longitude strata are listed, from west to east, in Tables S2 through S4 for Models 1 to 3, and estimates based on latitude strata, from south to north, are listed in Tables S5 through S7 for Models 1 to 3. The combined results in these tables did not change the pattern of results. All statistical analyses were performed using R version 3.2.2 (Foundation for Statistical Computing, Vienna, Austria). A complete description of the statistical methods is provided in Data S1 with estimated measures of covariates for each of the models (Tables S8 through S11).

## Results

Our cohort included a large fraction of US hemodialysis patients undergoing in‐center hemodialysis between January 2011 and December 2013. Over a third of patients were black, consistent with the high incidence of end‐stage renal disease in this community, and the average follow‐up time was ≈12 months (Table 1). Dialysate sodium levels dropped by 0.25 mEq/L (95% CI 0.24–0.26) and erythropoietin dose/treatment by 34.4%, suggestive of improved standards of care over the observation period.

**Table 1:** Patient Demographics, Clinical/Laboratory Parameters and Dialysis Treatment Data With Means and Standard Deviations

Variable	All (N=342 457) (100%)		Black (n=123 908) (36.2%)		White (n=218 549) (63.8%)	
	Mean	SD	Mean	SD	Mean	SD
Average follow‐up time, mo	12.51	12.16	14.05	12.85	11.64	11.67
Demographics						
Men, %	57		54		59	
Age, y	59.46	15.55	55.30	15.13	61.84	15.28
Body mass index, kg/m^2^	29.17	7.97	29.39	8.25	29.04	7.80
Existing hypertension, %	66.96		70.83		64.78	
Clinical data						
Pre‐dialysis SBP, mm Hg	146.84	20.72	149.87	20.36	145.13	20.72
Post‐dialysis SBP, mm Hg	137.79	18.79	139.97	18.80	136.55	18.66
Pre‐dialysis DBP, mm Hg	76.83	12.53	80.62	12.45	74.68	12.05
Post‐dialysis DBP, mm Hg	72.46	10.89	75.37	10.95	70.81	10.50
Pre‐dialysis weight, kg	84.73	23.56	86.49	24.32	83.73	23.06
Post‐dialysis weight, kg	82.36	23.06	84.03	23.81	81.41	22.57
Pre‐dialysis body temperature, °C	36.36	0.29	36.38	0.28	36.34	0.29
Post‐dialysis body temperature, °C	36.43	0.27	36.45	0.27	36.42	0.28
Laboratory data						
Serum sodium, mEq/L	138.38	2.84	138.93	2.61	138.05	2.92
Serum potassium, mEq/L	4.71	0.54	4.64	0.51	4.74	0.55
Hemoglobin, g/dL	10.80	0.96	10.80	0.96	10.81	0.96
Albumin, g/dL	3.75	0.45	3.80	0.43	3.73	0.45
Dialysis treatment data						
Central venous catheter as vascular access, %	24.65		20.85		26.80	
Treatment time, min	219.80	29.02	222.28	29.50	218.38	28.65
Ultrafiltration rate, mL/h per kg	8.43	3.40	8.51	3.27	8.39	3.48
Equilibrated, Kt/V	1.50	0.28	1.45	0.23	1.52	0.30
Interdialytic weight gain, kg	2.48	1.14	2.57	1.11	2.43	1.16
Erythropoietin dose (U/dialysis)	4178	4716	4419	4872	4042	4619

Differences between black and white patients are significant for all variables (all *P*<0.05). Monthly data from January 2011 to December 2013 were collected from each of 342 457 patients who underwent dialysis in 2177 Fresenius Medical Care North America facilities. These 2177 facilities correspond to 1925 zip codes and 1530 latitude and longitude location pairs, and 44 111 patients died during this period. DBP indicates diastolic blood pressure; SBP, systolic blood pressure

**Figure 1:**
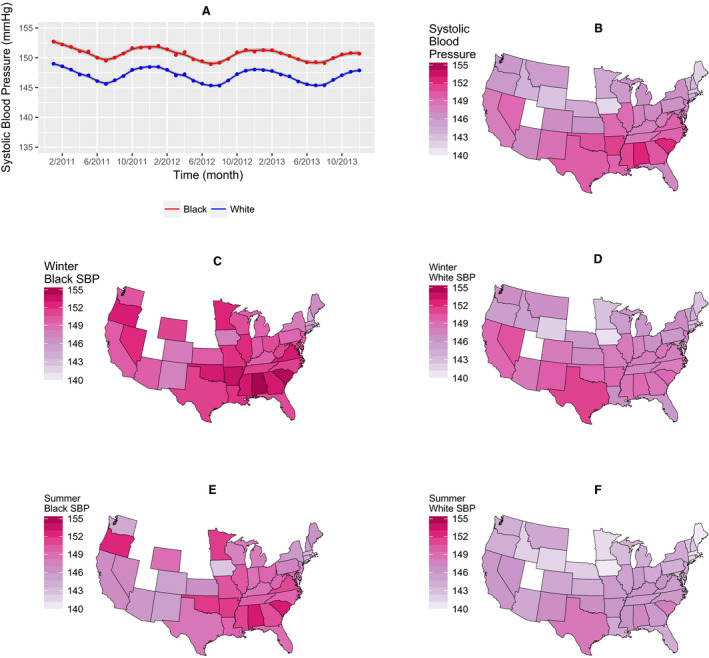
Seasonal, racial and geographical variation in blood pressure. **A**, Dots are monthly averages, lines are smoothing spline estimates, and shaded areas are 95% CI. Systolic blood pressure shows seasonal variation and is consistently higher in black than white patients. **B** through **F**, States with study centers and race‐specific SBP averages per state in winter and summer across the United States are shown. States with insufficient data (<10 patients or <2 months of observations) are shown in white. Mean annual SBP is highest in the “stroke belt” states (**B**). SBP is higher in winter (**C** and **D**) than summer (**E** and **F**) in all states. SBP is higher in black (**C** and **E**) than white (**D** and **F**) patients. SBP indicates systolic blood pressure.

**Figure 2:**
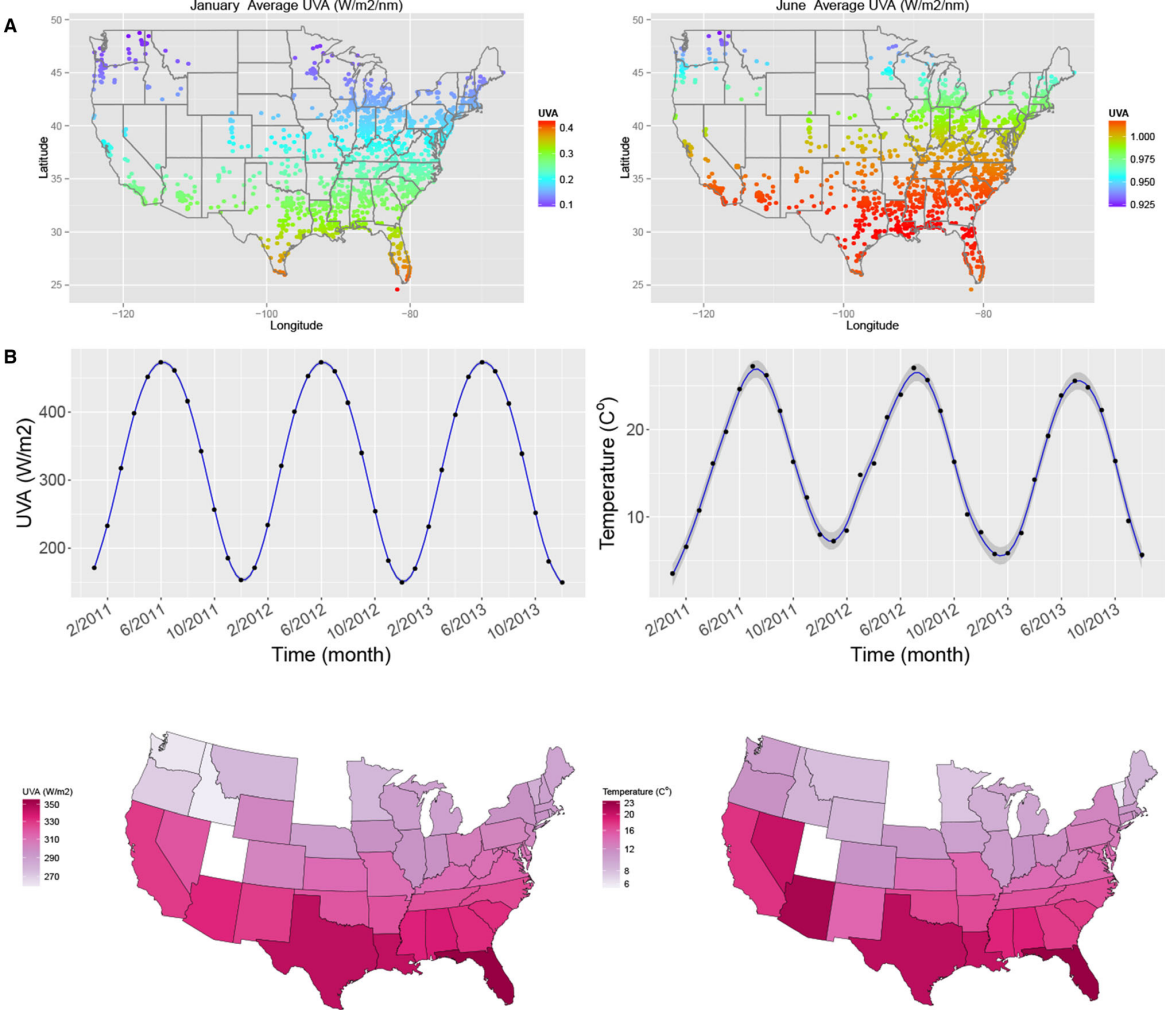
Outdoor exposure to solar UV light during a typical winter and summer month and seasonal variation of temperature and UV radiation. **A**, Exemplary monthly averages of UVA radiation in January and June 2011 by location (see Data S1 for monthly averages by wavelength band throughout that year). **B**, Seasonal variation in UVA exposure and temperature (shaded areas represent 95% CI), and annual averages by location.

**Figure 3:**
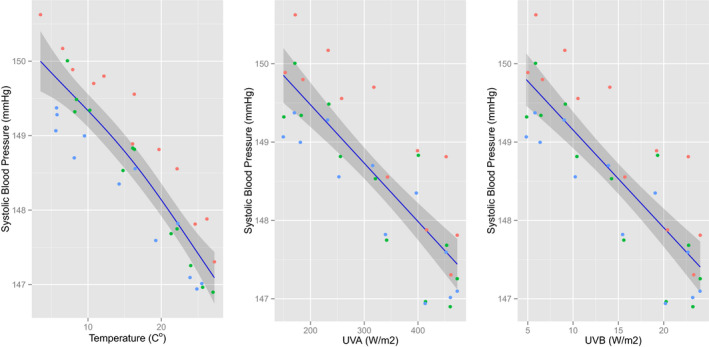
Association between environmental factors and systolic blood pressure. Temperature, UVA and UVB irradiation all inversely correlate with systolic blood pressure. Each dot represents mean nationwide UV for 1 calendar month. Red dots 2011, green dots 2012, blue dots 2013. Lines are smoothing spline estimates, and shaded areas are 95% CIs.

By computing monthly averages from a total of 45 784 963 individual BP measurements recorded over 36 months we observed clear seasonal variation in SBP (Figure 1A). Independent of this, there was a gradual fall of ≈1 mm Hg in SBP over the 3‐year period (Figure S5). SBP was ≈4 mm Hg higher in black than white dialysis patients. Mean annual SBP was highest in the south‐eastern states except Florida, mapping onto what is known as the “stroke belt” (Figure 1B). We next computed monthly averages of outdoor temperature and solar radiation (in the form of UV light) at each of the dialysis facilities using high‐fidelity daily and hourly records retrieved from public databases. Temperature, UVA and UVB all showed the expected seasonal variation and geographical distribution (Figure 2 and Figures S2 through S4). All 3 variables were linearly and inversely associated with measured SBP (Figure 3). Means (and SD) of temperature, UVA and UVB were 16.0 (9.02) °C, 320.9 (118.0) W/m^2^, and 14.7 (7.13) W/m^2^, respectively. Although UV and temperature were closely associated (correlation coefficients between UVA/UVB and temperature: 0.80/0.81), collinearity was deemed not to be a problem.

We next fitted 3 separate models to adjust for possible confounders. The SBP fall with increasing incident UV exposure was seen in Model 1 (unadjusted data) and Model 2 (with adjustment for covariates). Correcting for temperature (in Model 3) did not alter the statistically significant inverse relationship between UV and SBP, albeit less strongly than before (Table 2). The fall in SBP for a given rise in energy was greater for UVB than UVA, and larger in whites than blacks.

**Table 2:** Relationship Between Systolic Blood Pressure and UV Light as Well as Temperature, Stratified by Skin Color and Wavelength Band

Model	UV Spectrum	White		Black	
		SBP Change Per Unit	95% CIs	SBP Change Per Unit	95% CIs
1	UVA	−0.78	(−0.81 to −0.76)	−0.64	(−0.68 to −0.61)
	UVB	−13.24	(−13.69 to −12.79)	−10.77	(−11.32 to −10.21)
2	UVA	−0.75	(−0.78 to −0.72)	−0.63	(−0.66 to −0.59)
	UVB	−12.73	(−13.22 to −12.23)	−10.49	(−11.07 to −9.91)
3	UVA	−0.32	(−0.37 to −0.27)	−0.23	(−0.29 to −0.16)
	Temperature	−8.13	(−8.99 to −7.27)	−7.37	(−8.47 to −6.26)
	UVB	−5.63	(−6.48 to −4.78)	−4.17	(−5.26 to −3.08)
	Temperature	−7.92	(−8.78 to −7.07)	−7.04	(−8.15 to −5.94)

Units for UV and temperature are 100×mm Hg/(W×m^−2^) and 100×mm Hg/°C, respectively, where a unit of 1.0 represents a change of population blood pressure of 1 mm Hg for a change of incident UV of 100 W/m^2^ or a change of temperature of 100°C. Model 1, no adjustment; Model 2, adjusted for clinical covariates and comorbidities; Model 3, model 2 with additional adjustment for ambient temperature; see Methods for details. Racial differences for the effects of UV radiation on blood pressure were significant in all models. SBP indicates systolic blood pressure.

Secondary analyses with standardized UV and temperature further corroborated these results (Tables S12 and S13). When temperature was divided into 4 strata, the effects of UV appeared to be larger when it was warmer, at least in whites, ie, the same UV radiation energy was associated with greater BP reduction at elevated temperatures (Table S13). Taken together, our analyses indicate that pre‐dialysis SBP is affected by solar UV radiation, even after adjustment for the prevailing outdoor temperature.

## Discussion

We here demonstrate an inverse correlation between pre‐dialysis SBP and incident UV radiation in a large and diverse cohort of US hemodialysis patients. Seasonal variation in blood pressure has previously been demonstrated in hypertensive populations^17^ and in patients on hemodialysis.^28^ Until now, however, these variations were attributed to differences in temperature alone. The size of our cohort and the mapping of detailed environmental data onto geographical locations allowed us to differentiate between different seasonally varying environmental factors affecting BP. The major finding of our study is that UV radiation intensity is inversely associated with SBP and that this association is independent of (albeit possibly modified by) the ambient temperature. Interestingly, despite dominating fluid‐dependent effects on BP in hemodialysis patients, an association between incident solar UV radiation and pre‐dialysis SBP could be discerned. This may account for the recent observation that mortality is lower in dialysis patients on sunnier days.^29^ If these observations were confirmed in non‐dialysis subjects, this could be of considerable public health significance as lower population BP directly translates into reduced renal, cerebrovascular and cardiovascular disease and lower mortality. We hope that these results will invigorate the search for other potentially beneficial environmental factors contributing to health and wellbeing.

Many cellular and bodily functions are subject to diurnal and seasonal change.^30^ Seasonal variation of BP is a complex phenomenon^31^, ^32^ that has been attributed mainly to variations in temperature or humidity,^21^, ^28^ although these variables are compounded by measurement errors and show poor agreement between indoor/outdoor conditions during the heating season.^33^ The geographical diversity and large number of dialysis centers at which we have collected data has enabled us to clearly discriminate between temperature and UV exposure. Consistent with previous reports,^17^, ^21^, ^28^ we find that temperature does indeed correlate with BP, but independently of this, so does UV. Our measures relate to incident UV outdoors. There is some evidence that environmental UV correlates with personal UV exposure,^34^ but in our cohort of patients with chronic illness we expect that the variations of environmental radiation recorded are a significant overestimate of the personal exposure experienced. Window glass absorbs almost all of the UVB, letting only longer wavelength UVA, visible and infrared light penetrate. Nevertheless, we suspect that most UV radiation is received during periods of time spent outside and the actual radiation received will only be a small proportion of that incident at each study site. Moreover, recent studies have shown that hemodialysis patients in New York, NY, walk significantly more than those in Baton Rouge, LA.^35^ If such differences in physical activity levels were consistent across the northern/southern state divide, this would be a further factor that could reduce BP in the less sunny north and confound estimated UV‐induced BP reduction. In this context the strong and consistent inverse association between environmental UV and SBP we here identified is striking, particularly as dialysis patients are often frail and spend relatively little time outside.

Many factors other than UV radiation affect BP. A great number of patients included in our study will have received antihypertensive medication, and the effect of the dialysis procedure on SBP is well known. However, one would not expect the former to change by season nor the latter to vary by geographical location. We observed a gradual fall of ≈1 mm Hg in recorded pre‐dialysis SBP over the 3‐year study period. While it is difficult to attribute this declining SBP trend to a specific intervention, erythropoietin use declined, and dialysis providers strive to improve fluid management by reducing dialysate sodium levels. In small studies these interventions resulted in SBP reductions, in particular in hypertensive patients.^36^, ^37^ We observed that mean SBP throughout the year was higher in southeastern states, consistent with the observation that hypertension is more prevalent in this part of the United States 38 (Figure 1B), despite the relatively high incident sunlight there. This area is known as the “stroke belt” of America and has had the highest incidence of cerebrovascular and cardiovascular^39^ deaths in the country since the middle of the 20th century, although the reason(s) for the underlying hypertension remain(s) uncertain. Whatever their nature, these factors must be strong enough to outweigh the hypotensive effects of sunshine.

If exposure to sunlight does indeed lower BP, a biologically plausible mechanism must be invoked. The intensity and spectral balance of solar radiation varies geographically and is affected most by latitude, altitude, and season. UVA (320–400 nm), the predominant form of UV reaching the earth's surface, will partly pass through window glass and penetrate as deep as the dermis in skin. Although not absorbed by DNA (and thus not directly mutagenic), it is absorbed by other photosensitizers and consequently generates free radicals, producing oxidative stress. UVB (280–320 nm) is responsible for the photochemical synthesis of previtamin D3 from 7‐dehydrocholesterol, but is also a direct DNA mutagen. It makes up ≈5% of incident UV radiation, and is around 2 orders of magnitude more erythemogenic than UVA. Shorter wavelength UVB penetrates less well through the atmosphere, so is reduced more than UVA at low altitude and as the sun's elevation falls in winter at higher latitudes. Individuals with high measured vitamin D levels have lower BP, but meta‐analyses of vitamin D supplementation studies,^40^, ^41^ and Mendelian randomization studies^42^ both show that vitamin D has no effect on BP control or cardiovascular mortality. Vitamin D itself thus cannot account for any postulated BP‐lowering actions of sunlight, although it may be a marker for sunlight^43^ or outdoor activity, which is known to reduce BP.

We^24^ and others^44^ have described an alternative mechanism by which UV can lower BP, independently of vitamin D. Human skin contains significant stores of NO‐related products.^45^ These can be mobilized by UV irradiation^45^ and blue light.^46^ UV radiation induces mobilization of NO from these stores to the circulation, where it dilates resistance vessels^24^ and lowers BP.^24^, ^44^ Photorelaxation of arterial smooth muscle was first shown ex vivo by Furchgott half a century ago^47^ and is possibly mediated via the same mechanism. The immediate photorelaxation peak in mammalian aorta is at 335 nm, with a post‐depletion maximum at around 310 nm.^48^ In our cohort we found that a given energy of UVB was more potent than UVA at lowering BP as signified by the steeper dose‐response curve (Table 2, Figure 3). It would also appear that the same photic energy is more efficient at elevated outdoor temperatures, at least in whites (Table S7), which may indicate that UV radiation and temperature synergize in cleaving NO‐containing storage forms in the skin. This observation warrants further investigation, and is consistent with a recent report of outdoor temperature changes having a larger impact on BP in southern compared with northern US cities.^49^ Differential absorption of UV radiation by the atmosphere results in a higher UVB/UVA ratio in summer, most marked at higher latitudes, which combined with the greater incident solar energy will account for reduced summer BP.

The duration of action of the BP‐lowering effects of UV exposure is uncertain. Experimentally, a single exposure to UV lowers blood pressure for <1 hour in health volunteers,^24^ yet the population of patients studied here must have had a more sustained fall in blood pressure as it lasted long enough to be recorded even when not immediately preceded by sun exposure. One must therefore assume that the SBP records used in the present analysis do not reflect acute but instead capture residual UV‐induced effects, and that BP lowering by chronic environmental UV exposure is secondary to a sustained shift in a more fundamental regulatory circuit linked to cardiovascular regulation such as eg, systemic and/or vascular redox status. Human skin is essentially a “biological detector” for electromagnetic radiation, and even low doses of UV elicit “photooxidative stress”.^50^ This is a universal response of tissues to light that extends to other life forms including plants and unicellular organisms, but in humans it has mostly been studied in the context of skin aging/photodamage, inflammation and carcinogenesis. It is conceivable that redox changes in the skin trigger systemic antioxidant responses, but we neither have experimental evidence for such a mechanism nor are we aware of any studies that investigated the relationship between skin redox and cardiovascular regulation.

Irrespective of mechanism, mean SBP was higher and the age younger in black than white patients, reflecting the well‐documented higher incidence of hypertension and faster trajectory of renal function decline in blacks than whites.^51^ BP in whites showed a steeper response to UV than that in blacks. This may reflect constitutive skin pigmentation shielding UV‐induced hypotensive effects. Jablonski argues that constitutive pigmentation predominantly protects against UVA, while seasonally altering adaptive pigmentation protects against seasonal variation in UVB.^52^ Constitutively dark skin protects against UV‐induced DNA damage,^53^ and both UVA and UVB transmission to the dermis is blocked more effectively by black than white skin. The mean transmission in the black epidermis is typically 7.4% UVB and 17.5% UVA, but 29.4% and 55.5% respectively, for white epidermis.^54^ Apart from these considerations the blunted BP response in blacks may also be because of effects downstream of UV‐induced release and/or production mechanisms. Salt sensitivity is more common in blacks than whites,^55^ and reduced NO bioavailability secondary to increased oxidative stress may contribute to the higher susceptibility to endothelial dysfunction, stroke, heart failure, peripheral arterial disease, and disparities in life expectancy in this population.^56^, ^57^ In addition, socioeconomic factors and dietary habits contribute to the racial disparity in hypertension risk.^58^ While hypertension development is a multifactorial affair, the different effects of UV radiation on white and black patients strengthened the notion of a skin‐mediated direct UV effect, internally validating our main finding.

We suggested earlier that exposure to natural sunlight^23^ may be beneficial for health and lower BP for reasons other than temperature. Modern lifestyles/working habits often translate into spending much time under artificial indoor lighting conditions, a topic that appears to have lost its research appeal.^59^ This is unfortunate as sunlight is important for more than vitamin D production and the entrainment of chronobiological processes. Since life on earth developed under the influence of solar radiation, it is difficult to imagine that human physiology should be unable to cope with UV radiation; more likely, modern man is “out of sync” with this important environmental stressor, which is an important part of our exposome. We suggest including UV exposure measures in future cardiovascular epidemiological studies.^60^ Nowadays, direct sunlight exposure is discouraged for fear of skin cancer (since UV is also a mutagen, inappropriate episodic over‐exposure must be avoided). The present study results indicate that normal environmental UV radiation has a BP‐lowering effect in hemodialysis patients. If confirmed to also hold true in the general population low exposure to or avoidance of sunlight may be a new and modifiable risk factor for hypertension. Of relevance, enhanced sunlight exposure was found to be associated with lower all‐cause mortality in a large female Scandinavian cohort.^61^ A 20‐year follow‐up with competing‐risk analysis of the same cohort revealed that while a habit of active sun exposure translated into an increased risk of death from cancer the extended life expectancy of women with sun‐seeking behavior was related to a decrease in cardiovascular disease‐related mortality. Since increased SBP is the major risk factor for CVD mortality our data may provide a working hypothesis to explain these associations.^62^

The present study has strengths and limitations. Its strengths relate to the large and diverse patient population, managed according to a standard protocol, by a single large dialysis provider with measurements recorded by professional healthcare providers, allied to high‐quality National Center for Atmospheric Research/National Oceanic and Atmospheric Administration environmental data. Weaknesses include the lack of personal‐level UV exposure measurements, differences in BP monitoring devices, the absence of data on anti‐hypertensive therapy, BP in‐between dialysis visits, socioeconomic status and physical activity level of patients. Our conscious decision to omit diastolic BP from our analyses represents another study limitation. We caution that the data relate to a North‐American cohort and may not necessarily be relevant for other geographical locations and populations of distinct genetic heritage. We have mitigated the absence of individual data on anti‐hypertensive medication by including the diagnosis of hypertension as a covariate, as it is a proxy marker for medication. This would reduce the apparent effect size of UV. While circulating vitamin D levels have been used to estimate personal exposure to UV radiation in other studies this approach would not be suitable in hemodialysis patients many of whom receive cholecalciferol supplementation to prevent secondary hyperparathyroidism^63^ and the data showing that Vitamin D has no effect on cardiovascular outcomes are extensive and robust.^64^

Seasonal variation in blood pressure has been known for 40 years, but, to our knowledge, for the first time we show here that this occurs independently of temperature. The reduction in blood pressure is more marked with a rise in UVB than UVA, and in whites than black people. Dermatological concerns about the skin cancer inducing effects of UV radiation need to be balanced against the observed blood pressure lowering effects of sunlight, particularly given the greatly higher burden of disease caused by hypertension.

## Sources of Funding

We acknowledge support from the US National Science Foundation (DMS‐1507620), the Center for Scientific Computing at the California NanoSystems Institute, the UK Medical Research Council (G1001536) and the Faculty of Medicine, University of Southampton.

## Disclosures

Drs Maddux, Usvyat, and Kotanko hold stock in Fresenius Medical Care. Dr Kotanko receives author honoraria from UpToDate. Prof Feelisch and Dr Weller are members of the Scientific Advisory Board of AOBiome, LLC and Relaxsol, Ltd. The remaining authors have no disclosures to report.

## Supporting information


**Data S1.**
**Figure S5.** Seasonal variation in (pre‐dialysis) systolic blood pressure superimposed onto linear downward trend in African‐American and white hemodialysis patients.
**Table S1.** Percentages of All Variables With Missing Data
**Table S2.** Relationship Between Systolic Blood Pressure and Ultraviolet Light Stratified by Race and Temperature and Longitude Based on Model 1
**Table S3.** Relationship Between Systolic Blood Pressure and Ultraviolet Light Stratified by Race and Longitude Based on Model 2
**Table S4.** Relationship Between Systolic Blood Pressure and Ultraviolet Light Stratified by Race and Longitude Based on Model 3
**Table S5.** Relationship Between Systolic Blood Pressure and Ultraviolet Light Stratified by Race and Latitude Based on Model 1
**Table S6.** Relationship Between Systolic Blood Pressure and Ultraviolet Light Stratified by Race and Latitude Based on Model 2
**Table S7.** Relationship Between Systolic Blood Pressure and Ultraviolet Light Stratified by Race and Latitude Based on Model 3
**Table S8.** Estimated Coefficients From Model 2, Stratified by UV Wavelength Band and Race
**Table S9.** Estimated Coefficients From Model 3, Stratified by Wavelength Band and Race
**Table S10.** Estimated Coefficients From Model 2 With an Extra Term of AGE^2^, Stratified by Wavelength Band and Race
**Table S11.** Estimated Coefficients From Model 3 With an Extra Term of AGE^2^, Stratified by Wavelength Band and Race
**Table S12.** Relationship Between Systolic Blood Pressure (SBP) and *Standardized* Ultraviolet Light as Well as *Standardized* Temperature, Stratified by Race and Wavelength Band
**Table S13.** Relationship Between Systolic Blood Pressure (SBP) and Ultraviolet Light, Stratified by Race and Temperature
**Figure S1.** Geographical location of dialysis facilities at which blood pressure data were collected (**A**) and spatial distribution of patient numbers by State (**B**).
**Figure S2.** Average UVA (W/m^2^) in the 4 seasons.
**Figure S3.** Average UVB (W/m^2^) in the 4 seasons.
**Figure S4.** Average temperature (°C) in the 4 seasons.
