# Supplementary material for: Does Incident Solar Ultraviolet Radiation Lower Blood Pressure?
Source: J Am Heart Assoc. 2020 Feb 28;9(5):e013837. doi: 10.1161/JAHA.119.013837 (PMC7335547; doi:10.1161/JAHA.119.013837)
Supplement: Supplementary file 1 — Data S1. Figure S5. Seasonal variation in (pre‐dialysis) systolic blood pressure superimposed onto linear downward trend in African‐American and white hemodialysis patients. Table S1. Percentages of All Variables With Missing Data Table S2. Relationship Between Systolic Blood Pressure and Ultraviolet Light Stratified by Race and Temperature and Longitude Based on Model 1 Table S3. Relationship Between Systolic Blood Pressure and Ultraviolet Light Stratified by Race and Longitude Based on Model 2 Table S4. Relationship Between Systolic Blood Pressure and Ultraviolet Light Stratified by Race and Longitude Based on Model 3 Table S5. Relationship Between Systolic Blood Pressure and Ultraviolet Light Stratified by Race and Latitude Based on Model 1 Table S6. Relationship Between Systolic Blood Pressure and Ultraviolet Light Stratified by Race and Latitude Based on Model 2 Table S7. Relationship Between Systolic Blood Pressure and Ultraviolet Light Stratified by Race and Latitude Based on Model 3 Table S8. Estimated Coefficients From Model 2, Stratified by UV Wavelength Band and Race Table S9. Estimated Coefficients From Model 3, Stratified by Wavelength Band and Race Table S10. Estimated Coefficients From Model 2 With an Extra Term of AGE2, Stratified by Wavelength Band and Race Table S11. Estimated Coefficients From Model 3 With an Extra Term of AGE2, Stratified by Wavelength Band and Race Table S12. Relationship Between Systolic Blood Pressure (SBP) and Standardized Ultraviolet Light as Well as Standardized Temperature, Stratified by Race and Wavelength Band Table S13. Relationship Between Systolic Blood Pressure (SBP) and Ultraviolet Light, Stratified by Race and Temperature Figure S1. Geographical location of dialysis facilities at which blood pressure data were collected (A) and spatial distribution of patient numbers by State (B). Figure S2. Average UVA (W/m2) in the 4 seasons. Figure S3. Average UVB (W/m2) in the 4 seasons. Figure S4. Average temperature (°C) in t [file JAH3-9-e013837-s001.pdf]

Supplemental Material

Data S1.

Additional Statistical Methods

Our goal was to investigate the relationship between SBP (systolic blood pressures) and UVA/UVB. Since repeated measurements of SBP from each patient at different locations were available, we considered a mixed effects model for the spatial-temporal data

$$y_i(s,t) = \beta_1(s) + b_{1i} + (\beta_2(s) + b_{2i})x(s, t) + \varepsilon_i(s,t), \quad s=1,\ldots,S; \quad t=1,\ldots,T, \qquad (1)$$

where $y_i(s,t)$ is the monthly average of pre-dialysis SBP of patient i in month t who underwent dialysis at location s, x(s, t) is the UVA (or UVB) at time t and location s, $\beta_1(s)$ and $\beta_2(s)$ are population intercept and slope at location s, b_{1i} and b_{2i} are random intercept and slope for patient i, $\varepsilon_i(s,t)$ is the random error, S=1350 is the total number of locations, and T=36 is the total number of months. We assume that the bivariate random vectors (b_{1i},b_{2i}) are independent and identically distributed (iid) Normal with mean zero and an unstructured covariance matrix, $\varepsilon_i(s,t)$ are iid Normal with mean zero and a constant variance, and random effects and random errors are mutually independent. We allow the population intercept and slope to depend on s since the mean SBP with zero UVA/UVB and association between SBP and UVA/UVB may depend on location. We may use a spatial model such as thin-plate spline for $\beta_1(s)$ and $\beta_2(s)$. However, the data set is too large to fit the spatial-temporal model to the whole data. For each fixed location s, model (1) is a simple linear mixed effect model which is feasible to fit. Therefore, we adopted a two-stage procedure:

1) fit a linear mixed effect model for each location s to get estimates $\widehat{\beta_2}(s)$ ‘s;
2) fit a thin-plate spline model to investigate spatial pattern and compute combined estimates of associations between SBP and UVA/UVB. We estimate and make inference of the association using a random effect model in meta-analysis. Specifically, under the assumption that $\widehat{\beta_2}(s)$ ‘s are independent among different locations, we consider a random effect model

$$\widehat{\beta_2}(s) = \beta_2 + \xi(s), \quad s = 1,\ldots,S,$$

where β_2 represents mean association between SBP and UVA/UVB, and $\xi(s)$ are iid Normal with mean zero and variance τ^2. Applying the DerSimonian and Laird procedure, we get the combined estimate of association

$$\widehat{\beta_2} = \left(\sum_{s=1}^{S} w(s)\right)^{-1} \left(\sum_{s=1}^{S} w(s)\ \widehat{\beta_2}(s)\right),$$

where

$$w(s) = \left(\hat{V}(s) + \widehat{\tau^2}\right)^{-1},$$

$\hat{V}(s)$ is the estimated variance of $\widehat{\beta_2}$(s),

$$\widehat{\tau^2} = \max\left\{0,\quad \frac{\sum_{s=1}^{S} \frac{\left(\widehat{\beta_2}(s) - \widetilde{\beta_2}\right)^2}{\hat{V}(s)} - (S-1)}{\sum_{s=1}^{S} \frac{1}{\hat{V}(s)} - \frac{\sum_{s=1}^{S} \frac{1}{\widehat{V^2}(s)}}{\sum_{s=1}^{S} \frac{1}{\hat{V}(s)}}}\right\}$$

and

$$\widetilde{\beta_2} = \frac{\sum_{s=1}^{S} \frac{\widehat{\beta_2}(s)}{\hat{V}(s)}}{\sum_{s=1}^{S} \frac{1}{\hat{V}(s)}}$$

We note that the above two-stage procedure may be regarded as a divide and recombine (divide and conquer) procedure for the analysis of big data[2].

Covariates were not considered in model (1). Based on the literature and preliminary analyses, we consider the following covariates: race, gender, age, comorbidity of hypertension, catheter use, BMI, IDWG, albumin, EPO dosage, hemoglobin, serum sodium and potassium. The ¼-power transformation is used for the EPO dosage. We have found that there is a downward linear trend of SBP during 2011-2013 (Figure S5). Furthermore, our main interest was to investigate if associations between SBP and UVA/UVB still existed after adjusting for temperature. Therefore, we considered the following three analyses:

i. Model 1: fit model (1) using two-stage analysis without adjustment for covariates.
ii. Model 2: fit model (1) with covariates race, gender, age, comorbidity of hypertension, catheter use, BMI, IDWG, albumin, EPO dosage, hemoglobin, serum sodium, potassium and a linear trend for calendar time in the first stage, and then combine estimated slopes using the DerSimonian and Laird procedure.

iii. Model 3: fit model (1) with all covariates as in (ii) and an additional covariate of temperature in the first stage, and then combine estimated slopes using the DerSimonian and Laird procedure.

We fitted linear mixed effects models using the *lme* function in the *nlme* R package with the restricted maximum likelihood (REML) method to estimate variance-covariance parameters. Rohrscheib et al[3] found that the relationship between blood pressure and age has an inverted "U"-shape for hemodialysis patients. Therefore, we conducted a sensitivity analysis where a quadratic function of age was added to Models 2 and 3. Estimated coefficients are listed in Tables S3A and S4A. Relationships between SBP and UVA/UVB are almost identical as those in Tables S1A and S2A. Therefore, conclusions remain the same. We had performed diagnostics to evaluate the model assumptions. We had also conducted other sensitivity analyses (not presented) and the conclusions remain the same.

Figure S5. Seasonal variation in (pre-dialysis) systolic blood pressure superimposed onto linear downward trend in African-American and White hemodialysis patients.

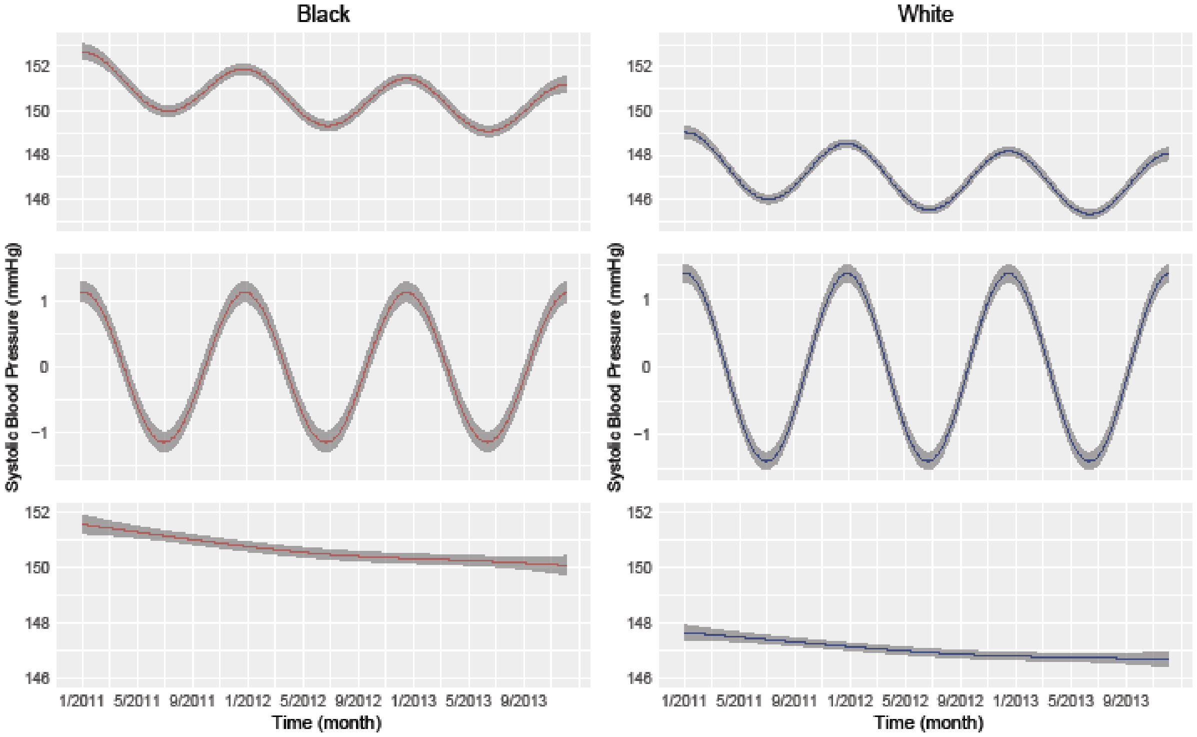

Top panels are partial spline fits to monthly averages of SBP. Middle and bottom panels are, respectively, the seasonal trend and long-term trend over calendar time based on the partial spline fits. Left and right panels correspond to Black and White patients. There is a clear downward linear trend over calendar time which is included in Models 2 and 3. We attribute this downward trend to improved standards of care over the observation period.

Table S1. Percentages of all variables with missing data.

	All (N=342,457)	Black (N=123,908) (36.2%)	White (N=218,549) (63.8%)
**Variables**	Missing %	Missing %	Missing %
SBP	5.61	5.12	5.94
Albumin (g/dL)	8.38	7.60	8.91
Age (years)	0.91	0.75	1.01
Interdialytic weight gain (kg)	1.61	1.48	1.70
Serum sodium (mEq/L)	15.89	13.69	17.42
Body mass index (kg/m^2)	7.71	6.35	8.64
Serum potassium (mEq/L)	7.87	7.31	8.26
Hemoglobin (g/dL)	4.19	3.85	4.42
Temperature (°C)	18.05	16.58	19.06

Table S2. Relationship between systolic blood pressure and ultraviolet light stratified by race and temperature and longitude based on Model 1.

Longitude	UV spectrum	White		Black	
		SBP change per unit UV	95% Confidence intervals	SBP change per unit UV	95% Confidence intervals
**(-125,-110]**	*UVA*	-1.00	(-1.07 to -0.92)	-0.96	(-1.15 to -0.77)
	UVB	-16.78	(-17.99 to -15.57)	-15.94	(-19.06 to -12.82)
**(-110,-95.8]**	*UVA*	*-1.00*	*(-1.08 to -0.91)*	*-0.91*	*(-1.09 to -0.73)*
	UVB	*-16.25*	*(-17.60 to -14.90)*	*-14.70*	*(-17.59 to -11.81)*
**(-95.8,-81.4]**	*UVA*	*-0.69*	*(-0.73 to -0.65)*	*-0.62*	*(-0.68 to -0.56)*
	UVB	*-11.74*	*(-12.40 to -11.08)*	*-10.31*	*(-11.22 to -9.38)*
**(-81.4,-67]**	*UVA*	*-0.72*	*(-0.76 to -0.68)*	*-0.60*	*(-0.64 to -0.55)*
	UVB	*-12.37*	*(-13.10 to -11.64)*	*-10.10*	*(-10.90 to -9.30)*
**Combined**	*UVA*	*-0.78*	*(-0.81 to -0.76)*	*-0.64*	*(-0.68 to -0.61)*
	UVB	*-13.24*	*(-13.69 to -12.79)*	*-10.77*	*(-11.32 to -10.21)*

Table S3. Relationship between systolic blood pressure and ultraviolet light stratified by race and longitude based on Model 2.

Longitude	*UV spectrum*	White		Black	
		SBP change per unit UV	95% Confidence intervals	SBP change per unit UV	95% Confidence intervals
**(-125,-110]**	*UVA*	-1.05	(-1.13 to -0.97)	-1.17	(-1.35 to -0.99)
	UVB	-17.63	(-18.97 to -16.29)	-19.24	(-22.17 to -16.31)
**(-110,-95.8]**	*UVA*	-1.01	(-1.10 to -0.93)	-0.93	(-1.11 to -0.74)
	UVB	-16.52	(-17.91 to -15.13)	-15.06	(-17.98 to -12.14)
**(-95.8,-81.4]**	*UVA*	*-0.65*	*(-0.70 to -0.60)*	*-0.59*	*(-0.65 to -0.54)*
	UVB	*-10.99*	*(-11.75 to -10.23)*	*-9.87*	*(-10.78 to -8.97)*
**(-81.4,-67]**	*UVA*	*-0.66*	*(-0.70 to -0.61)*	*-0.56*	*(-0.61 to -0.51)*
	UVB	*-11.27*	*(-12.04 to -10.50)*	*-9.53*	*(-10.36 to -8.70)*
**Combined**	*UVA*	*-0.75*	*(-0.78 to -0.72)*	*-0.63*	*(-0.66 to -0.59)*
	UVB	*-12.73*	*(-13.22 to -12.23)*	*-10.49*	*(-11.07 to -9.91)*

Table S4. Relationship between systolic blood pressure and ultraviolet light stratified by race and longitude based on Model 3.

Longitude	UV spectrum	White		Black	
		SBP change per unit UV	95% Confidence intervals	SBP change per unit UV	95% Confidence intervals
**(-125,-110]**	*UVA*	-0.36	(-0.47 to -0.25)	-0.23	(-0.48 to 0.03)
	UVB	-6.05	(-7.84 to -4.26)	-3.88	(-8.08 to 0.32)
**(-110,-95.8]**	*UVA*	*-0.31*	*(-0.46 to -0.16)*	*-0.29*	*(-0.64 to 0.07)*
	UVB	*-5.39*	*(-7.80 to -2.99)*	*-4.89*	*(-10.52 to 0.74)*
**(-95.8,-81.4]**	*UVA*	*-0.34*	*(-0.43 to -0.24)*	*-0.25*	*(-0.36 to -0.14)*
	UVB	*-5.88*	*(-7.42 to -4.34)*	*-4.52*	*(-6.33 to -2.71)*
**(-81.4,-67]**	*UVA*	*-0.29*	*(-0.29 to -0.38)*	*-0.21*	*(-0.32 to -0.10)*
	UVB	*-5.25*	*(-6.86 to -3.63)*	*-3.86*	*(-5.69 to -2.03)*
**Combined**	*UVA*	*-0.32*	*(-0.37 to -0.27)*	*-0.23*	*(-0.29 to -0.16)*
	UVB	*-5.63*	*(-6.48 to -4.78)*	*-4.17*	*(-5.26 to -3.08)*

Table S5. Relationship between systolic blood pressure and ultraviolet light stratified by race and latitude based on Model 1.

Latitude	*UV spectrum*	White		Black	
		SBP change per unit UV	95% Confidence intervals	SBP change per unit UV	95% Confidence intervals
**(25.1,31.2]**	*UVA*	-1.00	(-1.09 to -0.91)	-0.78	(-0.89 to -0.68)
	UVB	-15.67	(-17.04 to -14.31)	-12.36	(-13.95 to -10.76)
**(31.2,37.3]**	*UVA*	-0.92	(-0.97 to -0.87)	-0.71	(-0.76 to -0.65)
	UVB	-14.99	(-15.77 to -14.20)	-11.57	(-12.46 to -10.67)
**(37.2,43.3]**	*UVA*	*-0.69*	*(-0.73 to -0.65)*	*-0.54*	*(-0.59 to -0.49)*
	UVB	*-11.94*	*(-12.55 to -11.32)*	*-9.40*	*(-10.24 to -8.56)*
**(43.3,49.4]**	*UVA*	*-0.56*	*(-0.63 to -0.48)*	*-0.66*	*(-1.30 to -0.02)*
	UVB	*-10.09*	*(-11.45 to -8.73)*	*-11.92*	*(-22.88 to -0.96)*
**Combined**	*UVA*	*-0.78*	*(-0.81 to -0.76)*	*-0.64*	*(-0.68 to -0.61)*
	UVB	*-13.24*	*(-13.69 to -12.79)*	*-10.77*	*(-11.32 to -10.21)*

Table S6. Relationship between systolic blood pressure and ultraviolet light stratified by race and latitude based on Model 2.

Latitude	*UV spectrum*	White		Black	
		SBP change per unit UV	95% Confidence intervals	SBP change per unit UV	95% Confidence intervals
**(25.1,31.2]**	*UVA*	-1.02	(-1.11 to -0.92)	-0.81	(-0.91 to -0.70)
	UVB	-15.91	(-17.41 to -14.41)	-12.72	(-14.42 to -11.01)
**(31.2,37.3]**	*UVA*	-0.87	(-0.93 to -0.82)	-0.70	(-0.76 to -0.64)
	UVB	-14.32	(-15.24 to -13.39)	-11.53	(-12.51 to -10.55)
**(37.2,43.3]**	*UVA*	*-0.64*	*(-0.68 to -0.60)*	*-0.50*	*(-0.55 to -0.46)*
	UVB	*-11.13*	*(-11.79 to -10.46)*	*-8.79*	*(-9.60 to -7.97)*
**(43.3,49.4]**	*UVA*	*-0.57*	*(-0.65 to -0.49)*	*-0.46*	*(-0.95 to 0.04)*
	UVB	*-10.31*	*(-11.75 to -8.87)*	*-8.28*	*(-17.19 to 0.62)*
**Combined**	*UVA*	*-0.75*	*(-0.78 to -0.72)*	*-0.63*	*(-0.66 to -0.59)*
	UVB	*-12.73*	*(-13.22 to -12.23)*	*-10.49*	*(-11.07 to -9.91)*

Table S7. Relationship between systolic blood pressure and ultraviolet light stratified by race and latitude based on Model 3.

Latitude	*UV spectrum*	White		Black	
		SBP change per unit UV	95% Confidence intervals	SBP change per unit UV	95% Confidence intervals
**(25.1,31.2]**	*UVA*	-0.39	(-0.578 to -0.20)	-0.06	(-0.29 to 0.17)
	UVB	-5.95	(-8.79 to -3.10)	-0.98	(-4.64 to 2.68)
**(31.2,37.3]**	*UVA*	-0.27	(-0.36 to -0.17)	-0.27	(-0.38 to 0.16)
	UVB	*-4.62*	*(-6.10 to -3.13)*	*-4.81*	*(-6,.61 to -3.01)*
**(37.2,43.3]**	*UVA*	*-0.34*	*(-0.42 to -0.26)*	*-0.25*	*(-0.36 to -0.15)*
	UVB	*-6.24*	*(-7.61 to -4.88)*	*-4.81*	*(-6.60 to -3.03)*
**(43.3,49.4]**	*UVA*	*-0.34*	*(-0.49 to -0.18)*	*-0.40*	*(-0.55 to -1.36)*
	UVB	*-6.28*	*(-8.95 to -3.61)*	*7.13*	*(-10.32 to 24.58)*
**Combined**	*UVA*	*-0.32*	*(-0.37 to -0.27)*	*-0.23*	*(-0.29 to -0.16)*
	UVB	*-5.63*	*(-6.48 to -4.78)*	*-4.17*	*(-5.26 to -3.08)*

Table S8. Estimated coefficients from Model 2, stratified by UV wavelength band and race.

Covariates	White		Black	
	SBP change per unit	95% Confidence intervals	SBP change per unit	95% Confidence intervals
UVA (W/m^2)	-0.0075	(-0.0078 to -0.0072)	-0.0063	(-0.0066 to -0.0059)
MALE	-3.7384	(-3.9844 to -3.4924)	-1.7899	(-2.1163 to -1.4634)
AGE (years)	-0.1942	(-0.2028 to -0.1855)	-0.0771	(-0.0893 to -0.0648)
EPO DOSE (U/dialysis)	0.2457	(0.2322 to 0.2592)	0.4317	(0.4121 to 0.4513)
ALBUMIN (g/dL)	4.6026	(4.4462 to 4.7591)	4.2375	(4.0367 to 4.4383)
INTERDIALYTIC WEIGHT GAIN (kg)	-0.8292	(-0.8938 to -0.7646)	-0.8260	(-0.9129 to -0.7391)
SERUM SODIUM (mEq/L)	0.1774	(0.1663 to 0.1885)	0.2222	(0.2081 to 0.2364)
BMI (kg/m^2)	0.2636	(0.2430 to 0.2843)	0.3004	(0.2729 to 0.3279)
SERUM POTASSIUM (mEq/L)	0.3972	(0.3403 to 0.4540)	0.2689	(0.1943 to 0.3435)
HYPERTENSION	5.3344	(5.0689 to 5.5999)	3.8018	(3.3122 to 4.2913)
HGB (g/dL)	1.1376	(1.0960 to 1.1792)	0.5007	(0.4517 to 0.5498)
CATHETER AS VASCULAR ACCESS	0.4708	(0.2910 to 0.6505)	0.6863	(0.4501 to 0.9225)
TIME (months)	-0.0553	(-0.0632 to -0.0474)	-0.0759	(-0.0857 to -0.0662)
UVB (W/m^2)	-0.1273	(-0.1322 to -0.1223)	-0.1049	(-0.1107 to -0.0991)
MALE	-3.7430	(-3.9894 to -3.4965)	-1.7873	(-2.1167 to -1.4579)
AGE (years)	-0.1941	(-0.2027 to -0.1855)	-0.0767	(-0.0892 to -0.0643)
EPO DOSE (U/dialysis)	0.2459	(0.2324 to 0.2593)	0.4317	(0.4121 to 0.4513)

ALBUMIN (g/dL)	4.6017	(4.4453 to 4.7582)	4.2356	(4.0347 to 4.4365)
INTERDIALYTIC WEIGHT GAIN (kg)	-0.8310	(-0.8956 to -0.7663)	-0.8268	(-0.9138 to -0.7398)
SERUM SODIUM (mEq/L)	0.1770	(0.1659 to 0.1881)	0.2217	(0.2075 to 0.2358)
BMI (kg/m^2)	0.2635	(0.2428 to 0.2843)	0.3008	(0.2733 to 0.3284)
SERUM POTASSIUM (mEq/L)	0.3981	(0.3412 to 0.4549)	0.2692	(0.1945 to 0.3439)
HYPERTENSION	5.3330	(5.0673 to 5.5988)	3.8205	(3.3253 to 4.3157)
HGB (g/dL)	1.1389	(1.0972 to 1.1805)	0.5011	(0.4520 to 0.5502)
CATHETER AS VASCULAR ACCESS	0.4717	(0.2919 to 0.6515)	0.6858	(0.4496 to 0.9220)
TIME (months)	-0.0548	(-0.0627 to -0.0469)	-0.0757	(-0.0854 to -0.0659)

The dose of erythropoietin (EPO DOSE) was transformed by raising it to the power of ¼.

Table S9. Estimated coefficients from Model 3, stratified by wavelength band and race.

	White		Black	
Covariates	SBP change per unit	95% Confidence intervals	SBP change per unit	95% Confidence intervals
UVA (W/m^2)	-0.0032	(-0.0037 to -0.0027)	-0.0023	(-0.0029 to -0.0016)
MALE	-3.6899	(-3.9412 to -3.4387)	-1.7891	(-2.0942 to -1.4841)
AGE (years)	-0.1913	(-0.2001 to -0.1825)	-0.0735	(-0.0846 to -0.0624)
EPO DOSE (U/dialysis)	0.2474	(0.2330 to 0.2618)	0.4322	(0.4115 to 0.4528)
ALBUMIN (g/dL)	4.4834	(4.3117 to 4.6551)	4.1338	(3.9217 to 4.3459)
INTERDIALYTIC WEIGHT GAIN (kg)	-0.8487	(-0.9205 to -0.7768)	-0.8207	(-0.9130 to -0.7285)
SERUM SODIUM (mEq/L)	0.1576	(0.1436 to 0.1717)	0.2104	(0.1963 to 0.2245)
BMI (kg/m^2)	0.2410	(0.2197 to 0.2623)	0.2788	(0.2508 to 0.3068)
SERUM POTASSIUM (mEq/L)	0.3763	(0.2761 to 0.4765)	0.2340	(0.1594 to 0.3087)
HYPERTENSION	5.3134	(5.0336 to 5.5931)	3.7728	(3.3570 to 4.1886)
HGB (g/dL)	1.1998	(1.1478 to 1.2519)	0.5527	(0.4999 to 0.6055)
CATHETER AS VASCULAR ACCESS	0.4515	(0.2582 to 0.6448)	0.6925	(0.4372 to 0.9477)
TIME (months)	-0.0470	(-0.0561 to -0.0379)	-0.0716	(-0.0828 to -0.0603)
TEMPERATURE (Celsius)	-0.0813	(-0.0899 to -0.0727)	-0.0737	(-0.0847 to -0.0626)
UVB (W/m^2)	-0.0563	(-0.0648 to -0.0478)	-0.0417	(-0.0526 to -0.0308)
MALE	-3.6862	(-3.9383 to -3.4340)	-1.7929	(-2.0984 to -1.4874)
AGE (years)	-0.1913	(-0.2002 to -0.1825)	-0.0735	(-0.0846 to -0.0625)

EPO DOSE (U/dialysis)	0.2472	(0.2328 to 0.2615)	0.4320	(0.4114 to 0.4527)
ALBUMIN (g/dL)	4.4852	(4.3142 to 4.6561)	4.1349	(3.9227 to 4.3472)
INTERDIALYTIC WEIGHT GAIN (kg)	-0.8490	(-0.9208 to -0.7773)	-0.8209	(-0.9132 to -0.7286)
SERUM SODIUM (mEq/L)	0.1573	(0.1444 to 0.1703)	0.2104	(0.1964 to 0.2245)
BMI (kg/m^2)	0.2411	(0.2198 to 0.2624)	0.2790	(0.2510 to 0.3070)
SERUM POTASSIUM (mEq/L)	0.3841	(0.3037 to 0.4644)	0.2339	(0.1592 to 0.3086)
HYPERTENSION	5.3053	(5.0242 to 5.5865)	3.7771	(3.3623 to 4.1919)
HGB (g/dL)	1.1954	(1.1475 to 1.2433)	0.5522	(0.4993 to 0.6051)
CATHETER AS VASCULAR ACCESS	0.4522	(0.2587 to 0.6457)	0.6936	(0.4382 to 0.9490)
TIME (months)	-0.0476	(-0.0566 to -0.0385)	-0.0720	(-0.0832 to -0.0608)
TEMPERATURE (Celsius)	-0.0792	(-0.0878 to -0.0707)	-0.0704	(-0.0815 to -0.0594)

The dose of erythropoietin (EPO DOSE) was transformed by raising it to the power of ¼.

Table S10. Estimated coefficients from Model 2 with an extra term of AGE^2, stratified by wavelength band and race.

Covariates	White		Black	
	SBP change per unit	95% Confidence intervals	SBP change per unit	95% Confidence intervals
UVA (W/m^2)	-0.0076	(-0.008 to -0.0073)	-0.0063	(-0.0067 to -0.0059)
MALE	-3.8402	(-4.1005 to -3.5799)	-1.9283	(-2.2605 to -1.596)
AGE (years)	-0.2079	(-0.2173 to -0.1984)	-0.1243	(-0.138 to -0.1106)
AGE^2 (years^2)	-0.0045	(-0.005 to -0.0041)	-0.005	(-0.0056 to -0.0045)
EPO DOSE (U/dialysis)	0.2464	(0.2314 to 0.2614)	0.4279	(0.4063 to 0.4496)
ALBUMIN (g/dL)	4.5825	(4.4072 to 4.7579)	4.303	(4.0771 to 4.5289)
INTERDIALYTIC WEIGHT GAIN (kg)	-0.8901	(-0.9637 to -0.8166)	-0.8074	(-0.9047 to -0.71)
SERUM SODIUM (mEq/L)	0.1647	(0.1521 to 0.1773)	0.2261	(0.2105 to 0.2418)
BMI (kg/m^2)	0.2204	(0.198 to 0.2428)	0.2781	(0.2504 to 0.3057)
SERUM POTASSIUM (mEq/L)	0.3918	(0.3289 to 0.4547)	0.2519	(0.1677 to 0.3361)
HYPERTENSION	5.2616	(4.9503 to 5.5729)	3.433	(3.0005 to 3.8655)
HGB (g/dL)	1.1452	(1.0991 to 1.1912)	0.5172	(0.4611 to 0.5732)
CATHETER AS VASCULAR ACCESS	0.5248	(0.3254 to 0.7242)	0.6851	(0.4213 to 0.9488)
TIME (months)	-0.0624	(-0.0714 to -0.0534)	-0.0781	(-0.0898 to -0.0663)
UVB (W/m^2)	-0.1292	(-0.1349 to -0.1235)	-0.1051	(-0.1118 to -0.0983)
MALE	-3.8414	(-4.1015 to -3.5813)	-1.9302	(-2.2625 to -1.5978)
AGE (years)	-0.2079	(-0.2174 to -0.1985)	-0.1243	(-0.138 to -0.1106)

AGE^2 (years^2)	-0.0046	(-0.0122 to 0.003)	-0.0048	(-0.0155 to 0.0059)
EPO DOSE (U/dialysis)	0.2465	(0.2315 to 0.2616)	0.4284	(0.4068 to 0.45)
ALBUMIN (g/dL)	4.5827	(4.4073 to 4.758)	4.3029	(4.077 to 4.5287)
INTERDIALYTIC WEIGHT GAIN (kg)	-0.8922	(-0.9658 to -0.8187)	-0.8088	(-0.9061 to -0.7114)
SERUM SODIUM (mEq/L)	0.1644	(0.1518 to 0.177)	0.2256	(0.21 to 0.2412)
BMI (kg/m^2)	0.2204	(0.1981 to 0.2428)	0.2782	(0.2506 to 0.3059)
SERUM POTASSIUM (mEq/L)	0.3925	(0.3296 to 0.4554)	0.2524	(0.1682 to 0.3365)
HYPERTENSION	5.2621	(4.951 to 5.5733)	3.4356	(3.0031 to 3.868)
HGB (g/dL)	1.146	(1.0999 to 1.1921)	0.5184	(0.4623 to 0.5744)
CATHETER AS VASCULAR ACCESS	0.5238	(0.3243 to 0.7232)	0.6835	(0.4197 to 0.9472)
TIME (months)	-0.0619	(-0.0709 to -0.0529)	-0.0778	(-0.0896 to -0.0661)

The dose of erythropoietin (EPO DOSE) was transformed by raising it to the power of ¼.

Table S11. Estimated coefficients from Model 3 with an extra term of AGE^2, stratified by wavelength band and race.

Covariates	White		Black	
	SBP change per unit	95% Confidence intervals	SBP change per unit	95% Confidence intervals
UVA (W/m^2)	-0.0031	(-0.0037 to -0.0026)	-0.0021	(-0.0028 to -0.0013)
MALE	-3.84	(-4.1012 to -3.5789)	-1.9035	(-2.2361 to -1.5708)
AGE (years)	-0.2087	(-0.2181 to -0.1993)	-0.124	(-0.1377 to -0.1103)
AGE^2 ($years^2$)	-0.0046	(-0.0051 to -0.0041)	-0.005	(-0.0056 to -0.0045)
EPO DOSE (U/dialysis)	0.2489	(0.2338 to 0.264)	0.4326	(0.4109 to 0.4544)
ALBUMIN (g/dL)	4.536	(4.3604 to 4.7117)	4.2458	(4.0193 to 4.4722)
INTERDIALYTIC WEIGHT GAIN (kg)	-0.8984	(-0.972 to -0.8249)	-0.8213	(-0.9188 to -0.7238)
SERUM SODIUM (mEq/L)	0.1565	(0.144 to 0.1691)	0.2197	(0.2041 to 0.2353)
BMI (kg/m^2)	0.2194	(0.1969 to 0.2419)	0.2756	(0.2478 to 0.3033)
SERUM POTASSIUM (mEq/L)	0.3863	(0.3236 to 0.4491)	0.2398	(0.1554 to 0.3241)
HYPERTENSION	5.259	(4.947 to 5.5711)	3.4428	(3.0094 to 3.8762)
HGB (g/dL)	1.1637	(1.1177 to 1.2097)	0.5419	(0.4858 to 0.5979)
CATHETER AS VASCULAR ACCESS	0.5173	(0.3177 to 0.7169)	0.6737	(0.409 to 0.9384)
TIME (months)	-0.0517	(-0.0609 to -0.0425)	-0.0712	(-0.0832 to -0.0592)
TEMPERATURE (Celsius)	-0.0849	(-0.0939 to -0.0759)	-0.0791	(-0.0914 to -0.0667)
UVB (W/m^2)	-0.0556	(-0.0646 to -0.0466)	-0.0384	(-0.0509 to -0.0259)
MALE	-3.8425	(-4.1034 to -3.5816)	-1.9065	(-2.2391 to -1.5738)

AGE (years)	-0.2087	(-0.2181 to -0.1993)	-0.124	(-0.1376 to -0.1103)
AGE^2 ($years^2$)	-0.0046	(-0.0051 to -0.0041)	-0.005	(-0.0056 to -0.0045)
EPO DOSE (U/dialysis)	0.2488	(0.2338 to 0.2639)	0.4327	(0.411 to 0.4544)
ALBUMIN (g/dL)	4.5366	(4.361 to 4.7123)	4.2482	(4.0218 to 4.4746)
INTERDIALYTIC WEIGHT GAIN (kg)	-0.8994	(-0.9729 to -0.8258)	-0.8215	(-0.919 to -0.724)
SERUM SODIUM (mEq/L)	0.1565	(0.144 to 0.1691)	0.2197	(0.2041 to 0.2353)
BMI (kg/m^2)	0.2194	(0.1969 to 0.2419)	0.2758	(0.2481 to 0.3035)
SERUM POTASSIUM (mEq/L)	0.3868	(0.3241 to 0.4495)	0.2406	(0.1563 to 0.325)
HYPERTENSION	5.2589	(4.9469 to 5.5708)	3.4449	(3.0116 to 3.8783)
HGB (g/dL)	1.1637	(1.1177 to 1.2098)	0.5416	(0.4855 to 0.5977)
CATHETER AS VASCULAR ACCESS	0.5167	(0.317 to 0.7163)	0.673	(0.4082 to 0.9377)
TIME (months)	-0.0519	(-0.0611 to -0.0428)	-0.0715	(-0.0835 to -0.0595)
TEMPERATURE (Celsius)	-0.0828	(-0.0918 to -0.0739)	-0.0756	(-0.0879 to -0.0632)

The dose of erythropoietin (EPO DOSE) was transformed by raising it to the power of ¼.

Table S12. Relationship between systolic blood pressure (SBP) and *standardized* ultraviolet light as well as *standardized* temperature, stratified by race and wavelength band.

Model	*UV band*	White		Black	
		SBP change per unit UV	95% Confidence intervals	SBP change per unit UV	95% Confidence intervals
1	*UVA*	-0.93	(-0.96 to -0.90)	-0.76	(-0.80 to -0.72)
	UVB	-0.94	(-0.97 to -0.91)	-0.74	(-0.80 to -0.72)
2	*UVA*	-0.90	(-0.94 to -0.86)	-0.74	(-0.78 to -0.70)
	UVB	-0.91	(-0.95 to -0.87)	-0.75	(-0.78 to -0.71)
3	*UVA*	-0.38	(-0.44 to -0.32)	-0.27	(-0.34 to -0.20)
	Temp	-0.73	(-0.81 to -0.65)	-0.67	(-0.77 to -0.56)
	UVB	-0.40	(-0.46 to -0.34)	-0.30	(-0.37 to -0.23)
	Temp	-0.71	(-0.79 to -0.63)	-0.64	(-0.74 to -0.53)

A standardized variable was calculated by subtracting mean and dividing by standard deviation of the original variable.

Table S13. Relationship between systolic blood pressure (SBP) and ultraviolet light, stratified by race and temperature.

Temp [ºC]	*UV band*	White		Black	
		SBP change per unit UV	95% Confidence intervals	SBP change per unit UV	95% Confidence intervals
<9.4	*UVA*	-0.51	(-0.61 to -0.42)	-0.03	(-0.16 to -0.09)
	UVB	-10.64	(-12.47 to -8.81)	-1.39	(-3.71 to -0.92)
9.4-17	*UVA*	-1.04	(-1.11 to -0.96)	-0.87	(-0.97 to -0.77)
	UVB	-16.94	(-18.28 to -15.60)	-14.40	(-16.08 to -12.72)
17-23.2	*UVA*	-0.97	(-1.06 to -0.89)	-0.61	(-0.72 to -0.50)
	UVB	-14.28	(-15.65 to -12.90)	-8.61	(-10.34 to -6.87)
>23.2	*UVA*	-2.21	(-2.35 to -2.08)	-0.34	(-0.52 to -0.16)
	UVB	-29.19	(-31.29 to -27.09)	-2.02	(-4.83 to 0.80)
Combined	*UVA*	-1.18	(-1.74 to -0.63)	-0.47	(-0.84 to -0.09)
	UVB	-17.73	(-24.15 to -11.32)	-6.67	(-12.72 to -0.61)

A linear model with all covariates in Model 3 is fitted to each stratum.

Figure S1. Geographical Location of Dialysis Facilities at which Blood Pressure Data were collected (panel A) and spatial distribution of patient numbers by State (panel B).

A

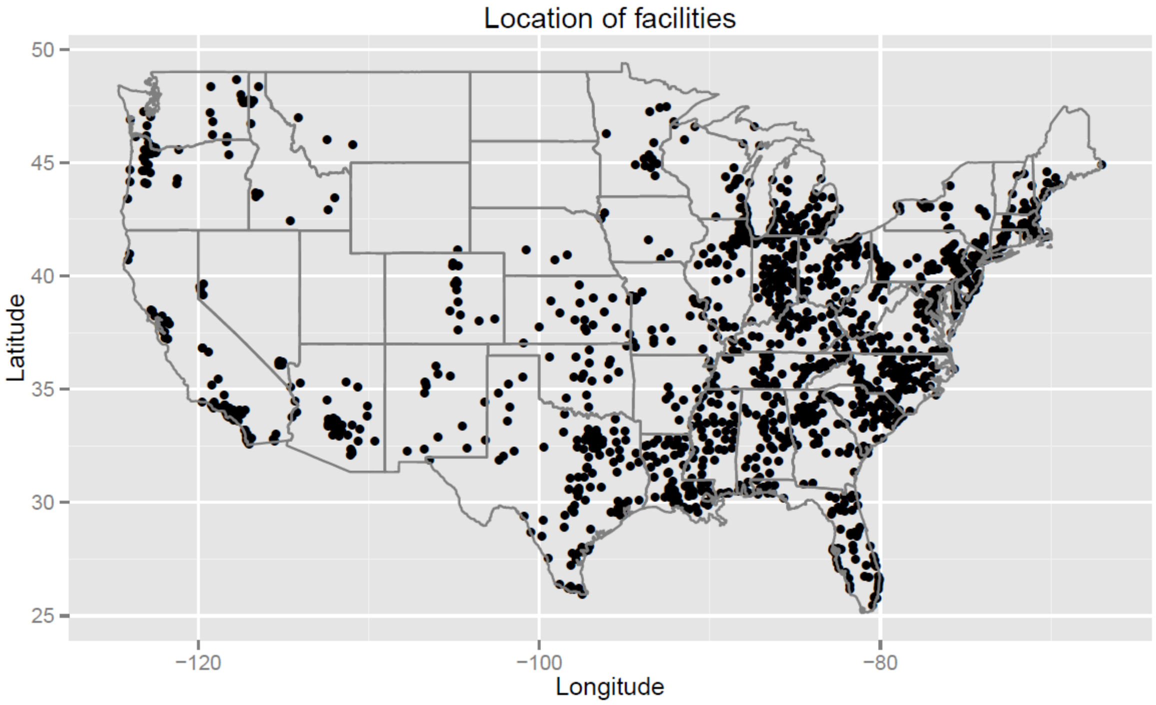

B

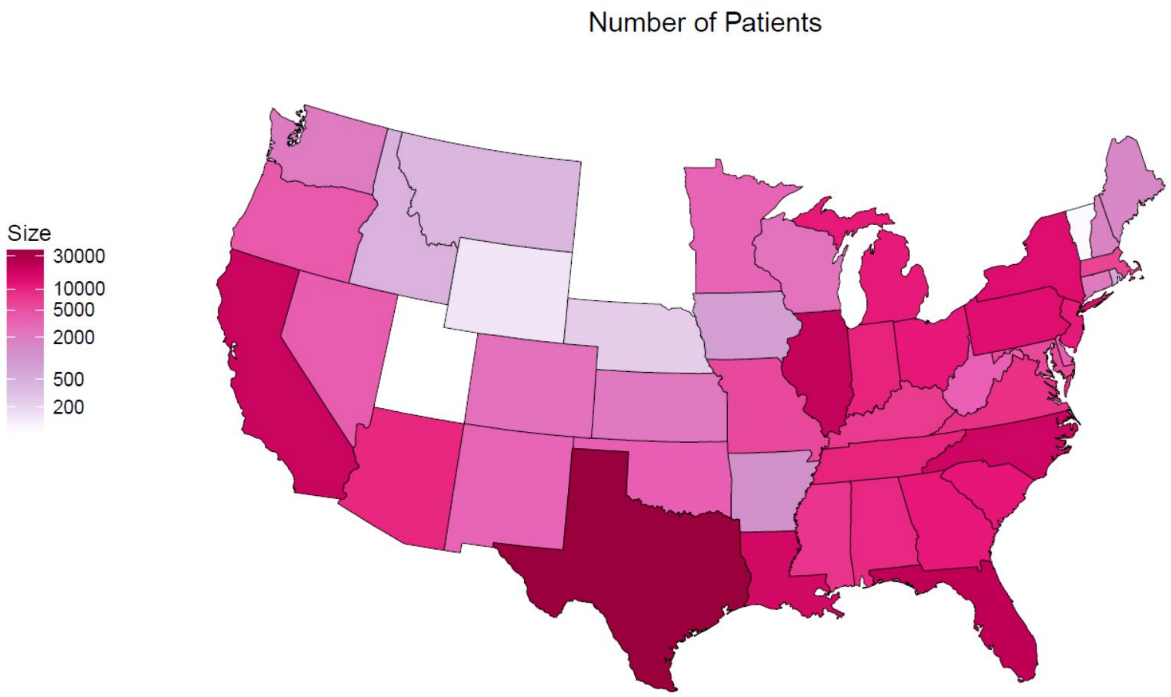

Figure S2. Average UVA (W.m^{-2}) in the four seasons.

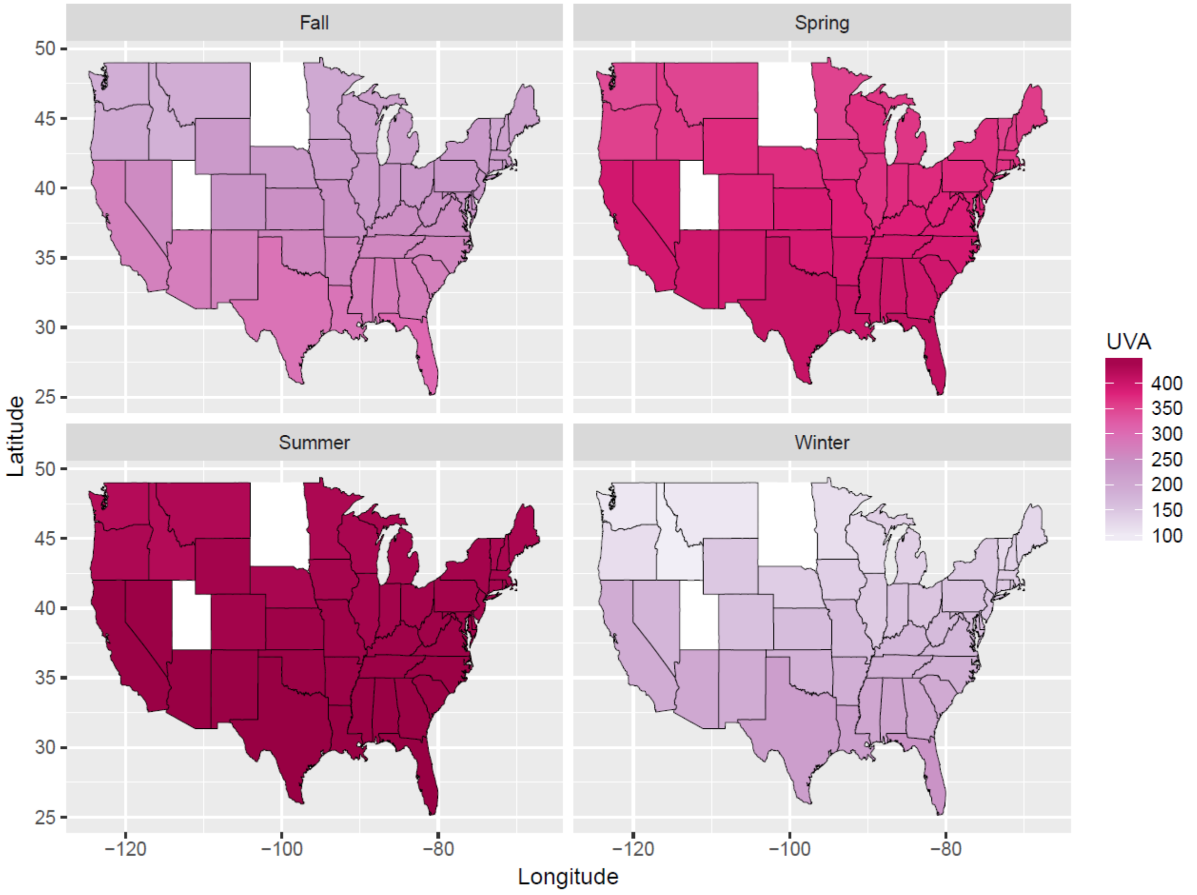

Figure S3. Average UVB ($W.m^{-2}$) in the four seasons.

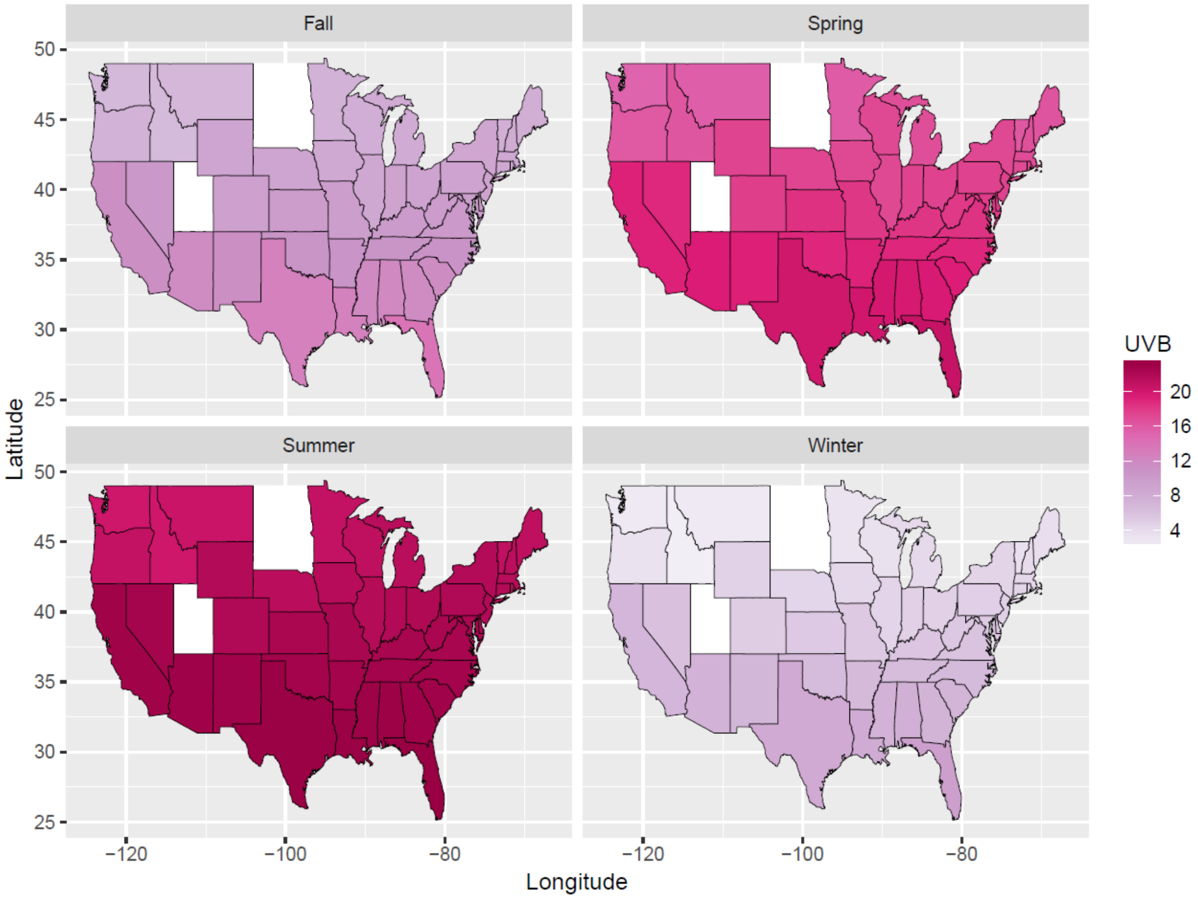

Figure S4. Average temperature (degree Celsius) in the four seasons.

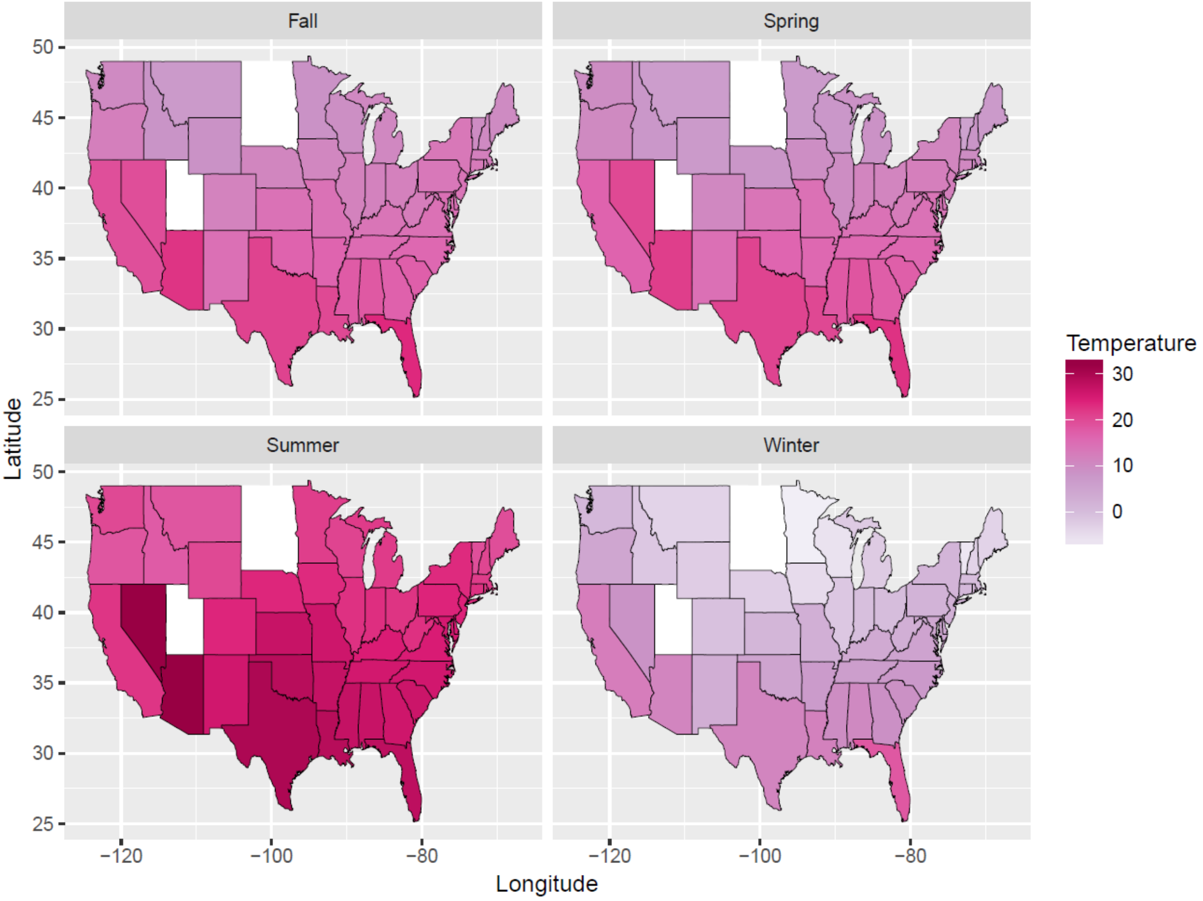

Supplemental References:

1. Wang, Y. Smoothing Splines: Methods and Applications. Champman and Hall: New York, 2011.
2. Guha S, Hafen R, Rounds J, Xia J, Li J, Xi B, Cleveland WG. Large complex data: divide and recombine (D&R) with RHIPE. Stat 2012; 1:53-67.
3. Rohrscheib MR, Myers OB, Servilla KS, Adams CD, Miskulin D, Bedrick EJ,Hunt WC, Lindsey DE, Gabaldon D, Zager PG for the DCI Medical Directors. Age-related Blood Pressure Patterns and Blood Pressure Variability among Hemodialysis Patients. Clin J Am Soc Nephrol 2008, 3: 1407-1414.
